# Stability assessment of hygroscopic medications in one dose packaging using community pharmacist surveys and experimental storage methods

**DOI:** 10.1038/s41598-025-89835-y

**Published:** 2025-02-18

**Authors:** Takayuki Yoshida, Hisashi Iijima, Yumeka Washio, Makoto Toda, Miho Morikawa, Kazuhiro Iwasa, Takashi Tomita

**Affiliations:** 1 Laboratory of Practical Community Collaboration, Center for Social Pharmacy, Teikyo Heisei University, 4-21-2 Nakano, Nakano-ku, Tokyo, 164-8530 Japan; 2 Drug Information Center, Chiba Pharmaceutical Association, 9-2 Tonyacho, Chuo-ku, Chiba-shi, Chiba, 260-0025 Japan; 3 Toshiba Nano-analysis Corporation, 1 Komukai Toshiba-Cho, Saiwai-ku, Kawasaki, 212-8583 Japan; 4 Department of Pharmaceutical Sciences, School of Pharmacy at Narita International University of Health and Welfare, 4-3 Kozunomori, Narita, 286-8686 Chiba Japan; 5 Department of Pharmacy, International University of Health and Welfare Mita Hospital, 1-4-3 Mita, Minato-ku, Tokyo, 108-8329 Japan

**Keywords:** One-dose package, Hygroscopic medication, Community pharmacist, Storage method, Health care, Health occupations, Medical research

## Abstract

Some medications are unsuitable for one-dose packaging due to issues such as moisture absorption. A survey of community pharmacists was conducted to understand the status of one-dose packaging and identify hygroscopic medications for stability testing. Stability was assessed using a moisture-suppression bag and clinical applications were investigated. Survey results were used to extract the medications that should be included in the one-dose package. Medications were stored with and without moisture-suppression bags, and quality was assessed using weight change and breaking force. Suvorexant, Telmisartan, Aspirin Dialuminate, Kampo extract formulations (Yokukansan and Daikenchu-tou), and Yokuinin extract tablets were selected for the stability test. Storage without moisture-suppression bags resulted in substantial changes in weight and breaking force for all tested medications; the use of moisture-suppression bags maintained the quality of some medications. Although there was a substantial difference in weight change from day 3, there was almost no change in appearance. Currently, the decision to include hygroscopic pharmaceuticals in a single package based on changes in appearance is insufficient. However, further research is necessary to justify the decision not to include hygroscopic pharmaceuticals in such packaging. Therefore, pharmaceutical companies should cultivate appropriate awareness and information literacy of pharmacists should be improved.

## Introduction

One-dose packaging is a method of dispensing two or more oral medications at different dosing points in a one-dose package, which helps improve compliance and adherence, especially in older adult patients, patients with hand and finger impairments, and those with impaired vision^1–3^. Since one-dose package dispensing involves dispensing unpackaged oral medications, degeneration becomes problematic when oral medications are stored in high humidity or exposed to light after dispensing^1^. Therefore, oral medications unsuitable for one-dose packaging are dispensed as press-through package (PTP) sheets or strip-packaged products^4^. Cellophane–polyethylene laminated and glassine papers, which are used to dispense non-packaged, oral medications in one-dose packaging dispensers, have low moisture resistance and light-shielding capabilities^5^. Therefore, degeneration of the oral medication may occur after one-dose packaging, depending on the packaging material. Pharmacists should be made aware of the material used for one-dose packaging and determine whether it is feasible to package oral medications in the same material for dispensing. However, it is difficult for pharmacists to immediately determine whether a medication is suitable for one-dose packaging at the time of dispensing, with reports of oral medications that are unsuitable for one-dose packaging being dispensed and subsequently being delivered to patients on cellophane-laminated polyethylene or glassine papers^6^.

The stability of pharmaceutical products is critical for ensuring clinical efficacy. Degradation of active ingredients can lead to reduced therapeutic effects, compromising patient outcomes. Furthermore, the Japanese Pharmacopoeia^7^, which sets quality standards for pharmaceutical products, specifies requirements for the mass and breaking force of formulations to ensure product reliability and patient safety. Compliance with these pharmacopeial guidelines is not only a regulatory obligation but also a key aspect of quality control in pharmaceutical practice. Increased humidity presents substantial challenges to the stability of hygroscopic medications, compromising both the physical and chemical stability of medications^8^ and potentially leading to microbial contamination^9^. Such contamination poses additional risks to patient health.

Addressing these stability challenges is particularly important for hygroscopic medications, which are more susceptible to environmental factors such as moisture. A study on hygroscopic tablets has shown that storing one-package preparations in polyethylene bags containing a desiccant prevents tablet weight gain due to moisture absorption and maintains tablet breaking force^10^. Matsuo et al. previously developed moisture-suppression bags and investigated the storage of one-dose packaged medications^11^. Although studies investigating post-dispensing storage conditions after dispensing one-dose packages for specific types of internal medications have been published, there is no research on tablets for which moisture absorption is a problem in insurance pharmacies, which in Japan, refers to community pharmacies accredited to dispense prescription medications under the national health insurance system.

Therefore, in the current study, we first administered a survey questionnaire to pharmacists who were members of the Chiba Prefecture Pharmaceutical Association to ascertain the actual status of one-dose packaged dispensing at insurance pharmacies. The reason for choosing the Chiba Pharmaceutical Association was that it has more than 2,000 members and is a region that has a mixture of urban and rural areas, so it was possible to gather a diverse range of opinions. This study aimed to address the challenges of one-dose packaging for hygroscopic pharmaceuticals in clinical settings. A survey questionnaire was conducted to gather insights into current dispensing practices and identify specific pharmaceuticals for further investigation. Based on the survey findings, we selected representative hygroscopic pharmaceuticals for stability testing. The stability tests were conducted as an independent component of this study using previously reported methods^8^, to evaluate optimal storage methods for these medications.

## Methods

### One-dose package dispensing survey

The survey was conducted through a web-based questionnaire using Microsoft Forms. A survey request form with a QR code was included in the April 2021 issue of the “Chiba Pharmaceutical Journal” (published on April 1, 2021), which was sent to 2060 members of the Chiba Pharmaceutical Association (as of March 31, 2021), and responses were invited. The survey period was 60 d, from April 1 to May 30, 2021, and responses were collected online. This survey was conducted with the approval of the Academic Ethics Review Committee of the Chiba Pharmaceutical Association (approval number: 20 − 05).

**Fig. 1:**
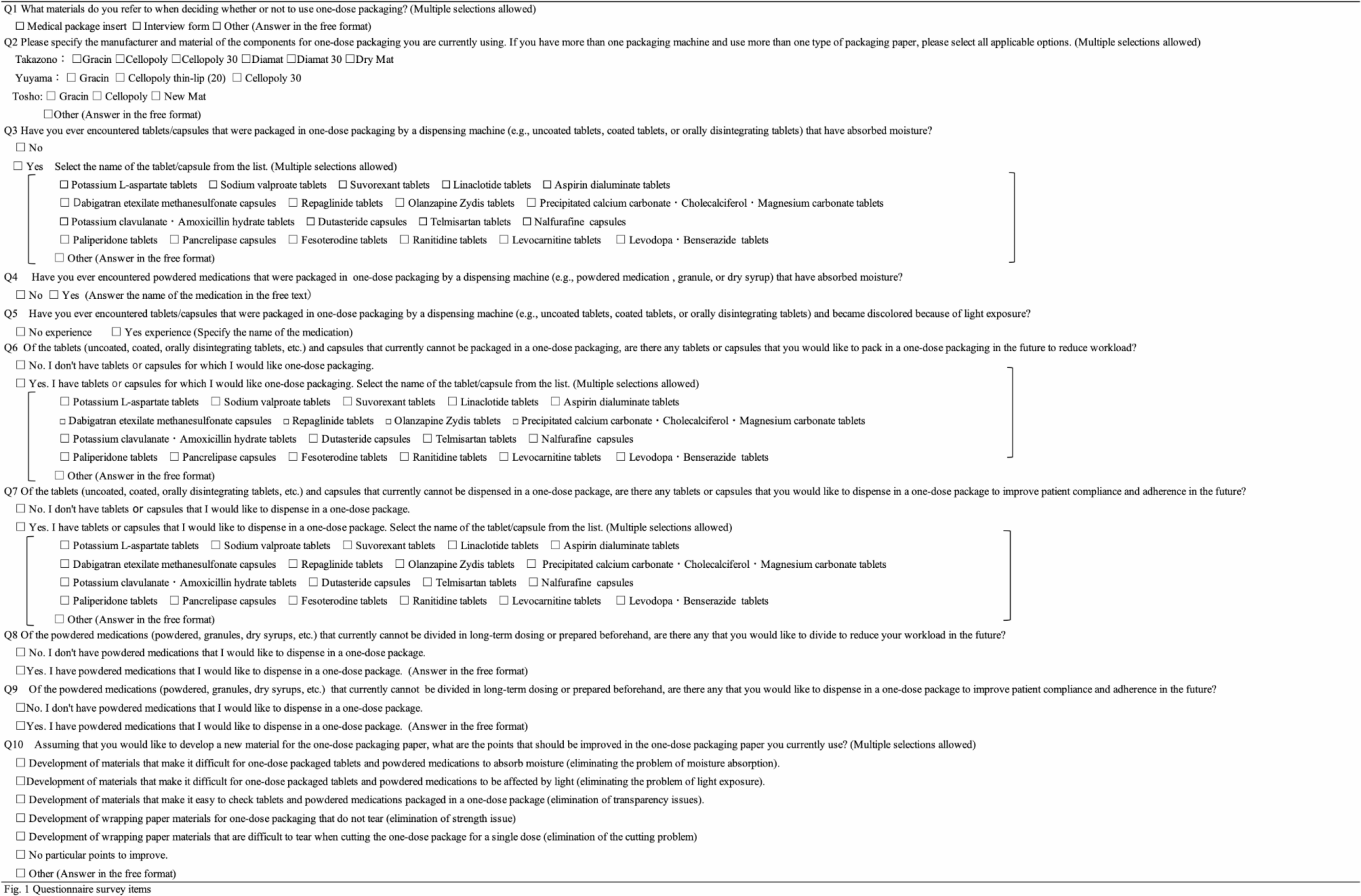
Items surveyed in a web-based questionnaire targeting members of the Chiba Pharmaceutical Association.

The survey items included information on materials used as references when deciding whether or not to use one-dose packaging. The term “interview form” used here refers to a document that provides information to supplement insufficient information in the package inserts prepared and provided by pharmaceutical companies (Q1). These questions followed: information on dispensing paper for one-dose packaging used in insurance pharmacies (Q2), information on dispensed medications for which moisture absorption or light exposure became a problem after being dispensed in a one-dose package (Q3–Q5), information on dispensed medications for which a one-dose package was desired (Q6–Q9), and requests for one-dose packaging dispensing paper (Q10) (Fig. 1). The names of the medications listed in the options for Q3, Q6, and Q7 were extracted from the “Pharmacy Case Study Search” of the “Project to Collect and Analyze Pharmaceutical Near-Miss Event Information” (http://www.yakkyoku-hiyari.jcqhc.or.jp/; last accessed January 6, 2025), which is available on the website of the Japan Council for Quality Health Care. The procedure for identifying cases in which hygroscopic medications were problematic was: from April 2009 to May 2020 (326,111 cases in total); 116 cases were extracted using the keywords “one-dose packaging” and “moisture absorption.” Among these, one-dose package dispensing was problematic in 102 cases. In one case, two medications were reported. There was a total of 103 medications, and the product names of the medications for which two or more drugs were reported were set as options. The data obtained from the questionnaires were tabulated.

### Selecting medications for storage experiments

**Fig. 2:**
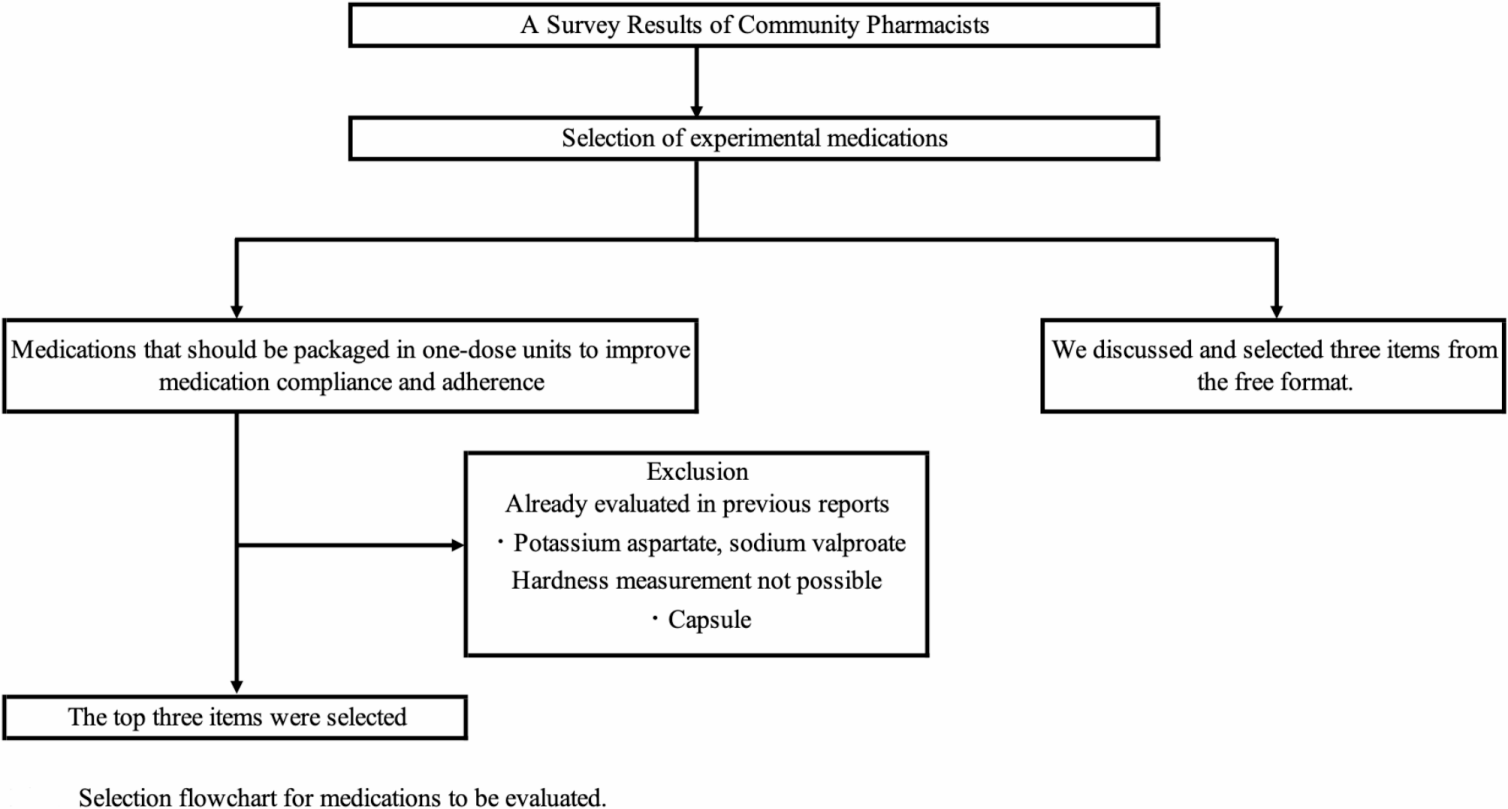
Selection flowchart for medications to be evaluated. indicating the process of selecting the medications to be evaluated from the survey results.

The selection in the survey questionnaire was based on the medications that pharmacists preferred to dispense in one-dose packages for compliance and adherence. The experimental medications were chosen from the top three items, excluding potassium L-aspartate tablets and sodium valproate tablets (which had been evaluated in previous reports), as well as capsule medications (which could not be measured for hardness). Furthermore, two experienced researchers selected three drugs from the list of medications that had been answered in the free format section (Fig. 2). The medications used for evaluation were the original medications.

### One-dose package methods

The machine used for packaging was Litrea II (Yuyama Co., Ltd., Osaka, Japan). Medications were packaged in 20-µm cellophane–polyethylene laminated paper (Yuyama Co., Ltd.) to accommodate a single dose.

### Moisture-suppression storage methods

Moisture-suppression bags described in a previous report^8^ were used for storage. The moisture-suppression bag had polyethylene terephthalate (12 μm), polyethylene (15 μm), aluminum films (7 μm) and polyethylene (15 μm) on the outside and a desiccant film (60 μm) on the inside (Kyushitsu-kun, Maruto Sangyo Co., Ltd., Fukuoka, Japan). The dimensions of the bags were 240 mm (width) × 270 mm (height) × 61.5 mm (thickness).

### Evaluation of the quality of hygroscopic medications

Stability testing was conducted to evaluate the effects of storage conditions on hygroscopic medications. Fifteen, one-dose packages of each product were prepared and divided equally into two storage conditions: (1) stored in moisture-suppression bags, and (2) stored without any protective packaging. The samples were stored under controlled conditions of temperature (35 ± 0.3 °C) and relative humidity (75 ± 2.5%) using a constant temperature and humidity chamber (PL-3KT, ESPEC Co., Ltd., Osaka, Japan). The weight of all packages was measured on day 0 to establish a baseline. Subsequently, five packages (*n* = 5) from each storage condition were removed at three timepoints (days 3, 7, and 14) to measure the rate of weight change. The weight change was calculated as the percentage difference from the baseline measurement.

The breaking force of the tablets was evaluated using a Kiya-type tablet breaking force tester (Fujiwara Manufacturing Co., Ltd., Tokyo, Japan) after measuring the weight. The measurement of tablet breaking force on day 0 was carried out using new tablets. The orientation of the tablets during measurement was standardized to ensure consistency across samples. For round tablets without a score line, the tablets were positioned such that compression was applied along their diameter. For tablets with a score line, the score line was oriented perpendicular to the compression plates. For irregularly shaped tablets, the tablets were positioned to apply the load along their diameter or the longest axis, depending on the tablet’s design. In this study, “d” is used as an abbreviation for “day” to indicate timepoints throughout the text. A sample size of five (*n* = 5) per timepoint was selected to balance experimental feasibility and statistical relevance. This sample size allows for reliable measurement of trends in weight and breaking force while minimizing the consumption of test samples. We decided to photograph the appearance of the tablets on the same background on days 3, 7, and 14.

### Statistical analysis

Simple tabulation of questionnaire data was performed to summarize the responses. No further statistical analyses were conducted on the questionnaire data, as it was not intended for in-depth statistical evaluation. The JMP Pro 18.1.0 software was used for analysis. The results are expressed as mean ± standard deviation. Statistical analysis was performed using one-way analysis of variance with the Tukey–Kramer test. Statistical significance was set at *P* < 0.05.

## Results

### Results of the survey questionnaire

Of the 2,060 pharmacists included in the study, 153 responded to the survey (response rate: 7.4%). Overall, 133 pharmacists (46.2%) referred to the package insert when deciding whether to use one-dose packaging and 95 pharmacists (33.0%) referred to the interview form. There were 60 respondents who selected “Other”. Of these, 38 (63.3%) indicated that they had contacted a pharmaceutical company (Table 1).

**Table 1:** Materials used as references in deciding whether or not to use one-dose packaging (*n* = 153) (multiple choice).

	Number of stated responses	(%)
Medical package insert	133	(46.2)
Interview form	95	(33.0)
Other	60	(20.8)
Total amount (%)	288	(100.0)

The most common material used for wrapping was cellophane–polyethylene laminated paper, which was also the most commonly used material for one-dose packaging. Of the 160 responses regarding material use, 75 (46.9%) were from Yuyama Co., Ltd., 52 (32.5%) were from Takazono Co., Ltd., Tokyo, Japan and 9 (5.6%) were from Tosho Co., Ltd., Tokyo, Japan Some respondents indicated the use of multiple materials (Table 2).

**Table 2:** Dispensing paper for one-dose packaging in insurance pharmacies (*n* = 153) (multiple choice).

Manufacturer name	Number of stated responses	(%)	Product name	Number of stated responses	(%)	Material	Number of stated responses	(%)
Yuyama Co., Ltd.	82	(49.1)	Cellopoly 30	60	(37.5)	Cellophane polyethylene Laminated paper	75	(46.9)
			Cellopoly thin-lip (20)	15	(9.4)			
			Glassine	7	(4.4)	Glassine	7	(4.4)
Takazono Co., Ltd.	68	(40.7)	Dia mat	35	(21.9)	Cellophane polyethylene laminated paper	52	(32.5)
			Dia mat30	4	(2.5)			
			Cellopoly	9	(5.6)			
			Cellopoly30	4	(2.5)			
			Dry mat	3	(1.9)	Polypropylene	3	(1.9)
			Glassine	13	(8.1)	Glassine	13	(8.1)
Tosho Co., Ltd.	10	(6.0)	Cellopoly	7	(4.4)	Cellophane polyethylene laminated paper	9	(5.6)
			New mat	2	(1.3)			
			Glassine	1	(0.6)	Glassine	1	(0.6)
Other	7	(4.2)						
Total amount (%)	167	(100.0)		160	(100.0)		160	(100.0)

Sixty (39.2%) pharmacists reported experiencing moisture absorption in tablets or capsules after dispensing them in one-dose packaging. Thirty-eight (24.8%) reported moisture absorption of powdered medications. Of these 38 people, 15 (39.5%) reported experience with Kampo extract formulations. These 15 people reported in the free description section that the most common brand was Yokukansan (4 cases) followed by Daikenchu-tou (2 cases). The tablets with the highest number of reports of moisture absorption were potassium l-aspartate tablets (27 cases; 33.3%), followed by sodium valproate tablets (22 cases; 27.2%). There was a high percentage of respondents who wanted one-dose packaging for each of these medications to reduce workload and improve medication compliance. In contrast, there was only one reported case (1.2%) of moisture absorption by suvorexant tablets. However, more than 10% of pharmacists preferred one-dose packaging to reduce workload and improve compliance and adherence (Table 3).

**Table 3:** Medication that absorbed moisture after one-dose packaging dispensing and medications for which one-dose packaging dispensing is desired (*n* = 153) (multiple choice).

	Medications that absorbed moisture after one-dose packaging dispensing		Medications for which one-dose packaging dispensing is desired			
	Number of stated responses (*n* = 60)	(%)	Reduction of workload		Improved compliance and adherence	
			Number of stated responses (*n* = 85)	(%)	Number of stated responses (*n* = 109)	(%)
Potassium L-aspartate tablets	27	(33.3)	46	(14.7)	54	(14.7)
Sodium valproate tablets	22	(27.2)	23	(7.3)	33	(9.0)
Aspirin・dialuminate tablets	5	(6.2)	20	(6.4)	28	(7.6)
Olanzapine Zydis tablets	2	(2.5)	7	(2.2)	9	(2.5)
Dutasteride capsules	2	(2.5)	25	(8.0)	24	(6.5)
Ranitidine tablets	2	(2.5)	8	(2.6)	8	(2.2)
Suvorexant tablets	1	(1.2)	35	(11.2)	43	(11.7)
Dabigatran etexilate methanesulfonate capsules	1	(1.2)	21	(6.7)	25	(6.8)
Potassium clavulanate・ Amoxicillin hydrate tablets	1	(1.2)	7	(2.2)	5	(1.4)
Levodopa・Benserazide tablets	1	(1.2)	20	(6.4)	22	(6.0)
Linaclotide tablets	0	(0.0)	12	(3.8)	13	(3.5)
Repaglinide tablets	0	(0.0)	7	(2.2)	11	(3.0)
Precipitated calcium carbonate・ Cholecalciferol・Magnesium carbonate tablets	0	(0.0)	9	(2.9)	12	(3.3)
Telmisartan tablets	0	(0.0)	22	(7.0)	27	(7.4)
Nalfurafine capsules	0	(0.0)	7	(2.2)	7	(1.9)
Paliperidone tablets	0	(0.0)	7	(2.2)	6	(1.6)
Pancrelipase capsules	0	(0.0)	6	(1.9)	6	(1.6)
Fesoterodine tablets	0	(0.0)	9	(2.9)	11	(3.0)
Levocarnitine tablets	0	(0.0)	6	(1.9)	8	(2.2)
Other	17	(21.0)	16	(5.1)	15	(4.1)
Total amount (%)	81	(100.0)	313	(100.0)	367	(100.0)

The most common request regarding one-dose packages pertained to the elimination of the problem of moisture absorption (115 cases; 33.0%), followed by light shielding (111 cases; 31.9%).

### Medications extracted from the questionnaire and those actually evaluated

Of the medications that the pharmacist preferred to dispense in one-dose packages for compliance and adherence, Suvorexant, aspirin dialuminate, and telmisartan tablets were selected. Of the medications sold in split or divided packages, the pharmacists indicated in the survey that they would like to redispense (open and remove the divided packages of drugs and repackage them by using a dispensing machine), Yokukansan and Daikenchu-tou Kampo extract formulations; hence, they were included in the survey. Yokuinin extract tablets (sold only in bottles) for which PTP products were not available and which absorbed moisture (as indicated in the survey questionnaire), were also included in the study.

Information on the one-dose packaged medications was: Bufferin Combination Tablets A81 (containing aspirin, dihydroxyaluminum amino acetate, and magnesium carbonate): 1 tablet/package (lot: 30441, Lion Co., Ltd., Tokyo, Japan), Belsomra Tablets 20 mg (containing suvorexant): 1 tablet/package (lot: X022144, MSD Co., Ltd., Tokyo, Japan), Micardis Tablets 40 mg (containing telmisartan): 1 tablet/package (lot: 389013, Japan Boehringer–Ingelheim Co., Ltd., Tokyo, Japan), Yokuinin extract tablets “KOTARO”: 3 tablets/package (lot: D2467, Kotaro Kampo Pharmaceutical Co., Ltd., Osaka, Japan), Daikenchu-tou Kampo extract formulation: 5 g/package (lot: W17112, Tsumura Co., Ltd., Tokyo, Japan), and Yokukansan Kampo extract formulation: 2.5 g/package (lot: W18052, Tsumura Co., Ltd.).

### Percentage change in the weights of various medications as a result of storage method

After 3 d of storage, the weight for medications that were not packaged in a moisture-suppression bag exhibited an increase of 1.3–7.2%, whereas that for the medications packaged in a moisture-suppression bag exhibited a decrease of 0.1% to an increase of 1.1%. Thus, the percentage change in weight was substantially less when the medications were stored in moisture-suppression bags. The percentage change in weight of Belsomra Tablets 20 mg after 3 d was approximately 1.1% for those stored in moisture-suppression bags and 7.2% for those not stored in moisture-suppression bags. Statistical analysis confirmed differences between the storage methods for all medications at 3 d.

- Bufferin Combination Tablets A81: F(1, 8) = 3617.577, *P* < 0.0001.
- Belsomra Tablets 20 mg: F(1, 8) = 36373.56, *P* < 0.0001.
- Micardis Tablets 40 mg: F(1, 8) = 912.6143, *P* < 0.0001.
- Yokuinin extract tablets: F(1, 8) = 121887.8, *P* < 0.0001.
- Daikenchu-tou Kampo extract formulation: F(1, 8) = 1335.831, *P* < 0.0001.
- Yokukansan Kampo extract formulation: F(1, 8) = 972.1678, *P* < 0.0001.

**Fig. 3:**
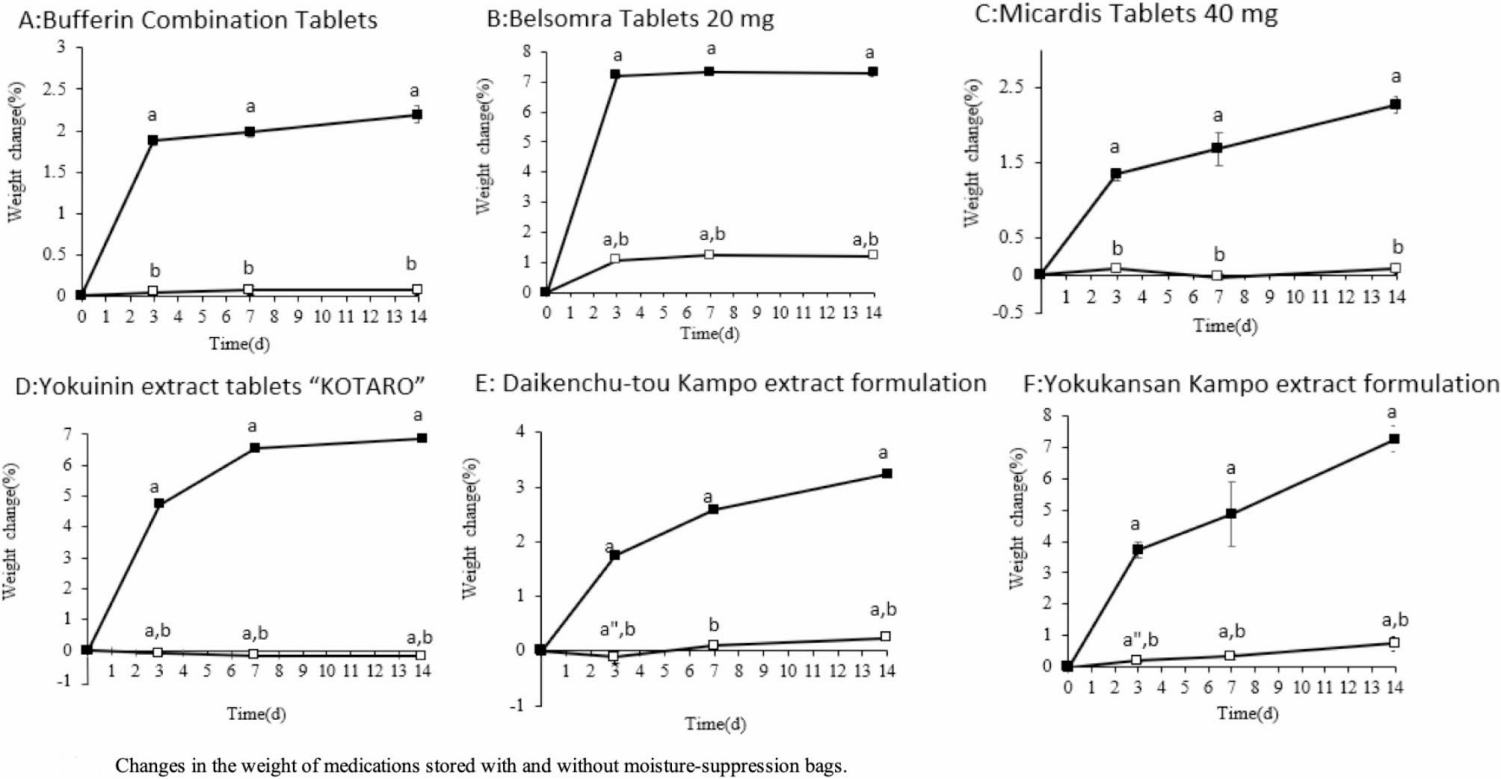
Changes in the weight of medications stored with and without moisture-suppression bags. Medications packed in cellophane–polyethylene laminating paper were stored with and without moisture-suppression bags. The samples were stored at 75% RH and 35 °C. Weight change (*n* = 5), Medications (**A**: Bufferin Combination Tablets A81, **B**: Belsomra Tablets 20 mg, **C**: Micardis Tablets 40 mg, **D**: Yokuinin extract tablets “KOTARO”, **E**: Daikenchu-tou Kampo extract formulation, and **F**: Yokukansan Kampo extract formulation); Storage method (filled square: without a moisture-suppression bag, unfilled square: with a moisture-suppression bag). ^a^*P* < 0.001, ^a”^*P* < 0.05 (vs. d 0), ^b^*P* < 0.001 (vs. storage without a moisture-suppression bag) (Tukey–Kramer test).

On comparing the medication weight on d 0 and the percentage change in weight, statistical differences were found for all medications stored without moisture-suppression bags. When stored in a moisture-suppression bag, no difference was observed for Bufferin Combination Tablets A81 only [(F(2, 12) = 0.7784, *P* = 0.5283)]. Although there were differences in the other medications, there were no differences in the post-hoc tests for Micardis 40 mg tablets at 3 d (*P* = 0.5824), 7 d (*P* = 0.9466), or 14 d (*P* = 0.5337) (Fig. 3). The following comparisons showed no differences:

- Belsomra Tablets 20 mg: 7 d vs. 14 d (*P* = 0.8740).
- Yokuinin extract tablets: 7 d vs. 14 d (*P* = 0.2833).
- Yokukansan Kampo extract formulation: 3 d vs. 7 d (*P* = 0.0663).

### Changes in breaking force by storage method

When stored in the moisture-suppression bag, breaking force changes were less than when the moisture-suppression bag was not used. Statistical analysis confirmed differences between the storage methods for all medications at 3 d:

- Bufferin Combination Tablets A81: F(1, 8) = 0.0393, *P* = 0.0393.
- Belsomra Tablets 20 mg: F(1, 8) = 25.1945, *P* = 0.0010.
- Micardis Tablets 40 mg: F(1, 8) = 43.5062, *P* = 0.0002.
- Yokuinin extract tablets: F(1, 8) = 217.9211, *P* < 0.0001.

**Fig. 4:**
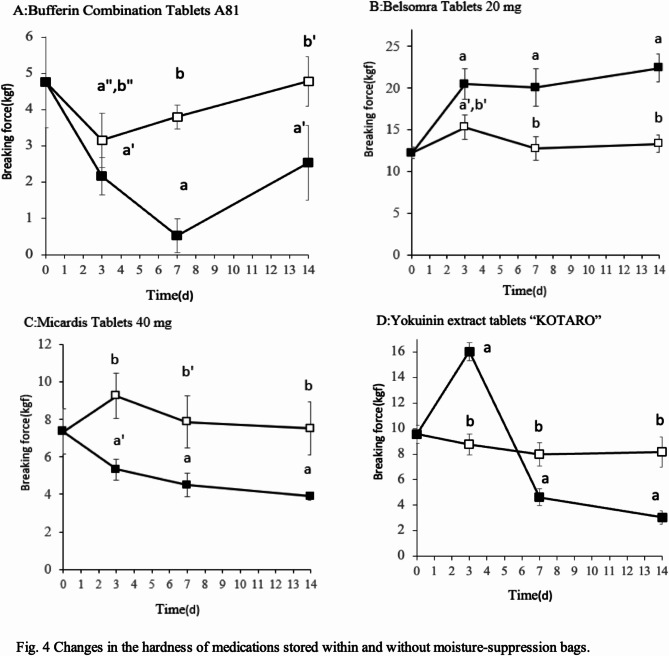
Changes in the breaking force of medications stored with and without moisture-suppression bags. Medications packed in cellophane–polyethylene laminating paper were stored with and without moisture-suppression bags. The samples were stored at 75% RH and 35 °C. Breaking force change (*n* = 5), Medications (**A**: Bufferin Combination Tablets A81, **B**: Belsomra Tablets 20 mg, **C**: Micardis Tablets 40 mg, **D**: Yokuinin extract tablets “KOTARO”); Storage method (filled square: without a moisture-suppression bag, unfilled square: with a moisture-suppression bag). ^a^*P* < 0.001, ^a’^*P* < 0.01 (vs. d 0), ^b^*P* < 0.001, ^b’^*P* < 0.01, ^b”^*P* < 0.05 (vs. storage without a moisture-suppression bag) (Tukey–Kramer test).

When not placed in a moisture-suppression bag, Belsomra Tablets 20 mg showed an increase in breaking force over the entire period, whereas Yokuinin extract tablets “KOTARO” showed an increase in breaking force only at 3 d. Other medications showed a decrease in breaking force (Fig. 4). When comparing the breaking force of the medications at d 0 with the breaking force on each day, a difference was observed in all drugs that were stored without using a moisture-suppression bag. When stored in a moisture-suppression bag, no differences were observed for Micardis Tablets 40 mg [F(3, 16) = 2.1899, *P* = 0.1290] and Yokuinin extract tablets [F(3, 16) = 2.9618, *P* = 0.0637].

### Changes in appearance of various medications by storage method

**Fig. 5:**
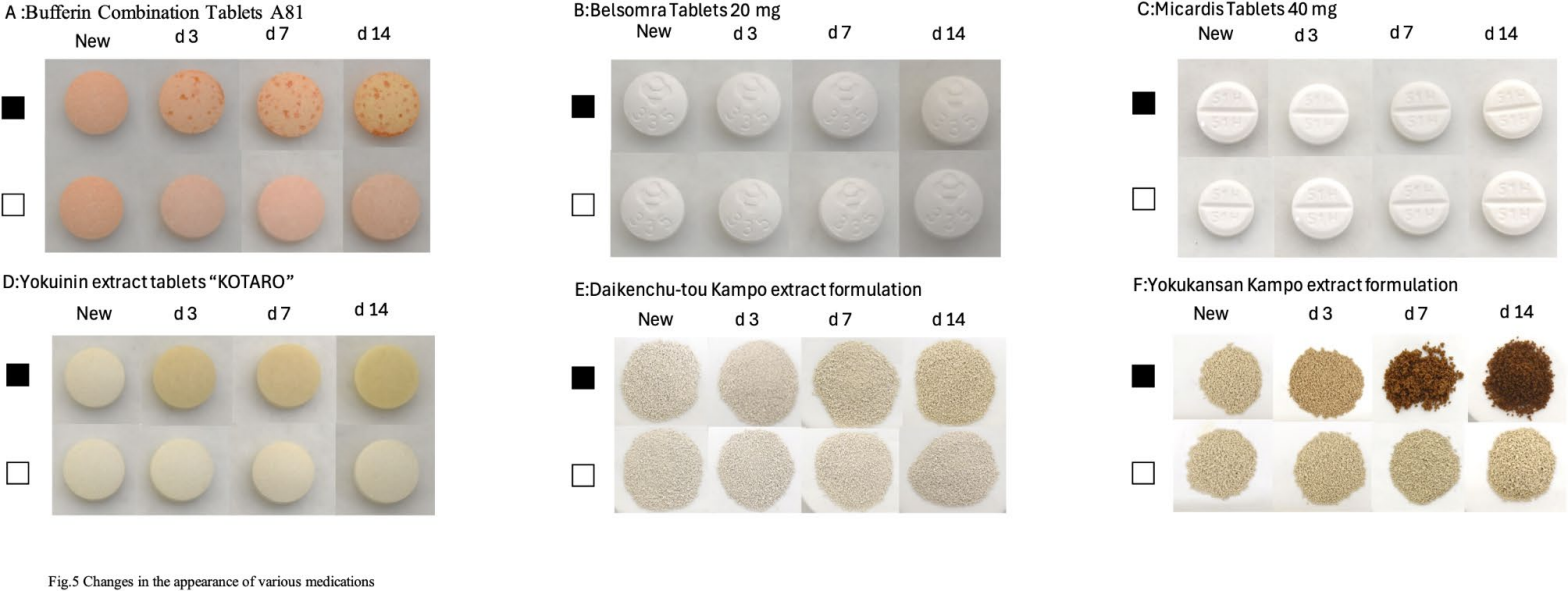
Changes in the appearance of various medications. The medications were stored at 75% RH and 35 °C. The appearance at 3, 7, and 14 d is shown. Medications (**A**: Bufferin Combination Tablets A81, **B**: Belsomra Tablets 20 mg, **C**: Micardis Tablets 40 mg, **D**: Yokuinin extract tablets “KOTARO”, **E**: Daikenchu-tou Kampo extract formulation, **F**: Yokukansan Kampo extract formulation); Storage method (filled square: without moisture-suppression bag; unfilled square: with moisture-suppression bag). The colored figure can be accessed in the online version.

Bufferin Combination Tablets A81, Yokuinin extract tablets “KOTARO,” and Yokukansan Kampo extract formulation showed obvious changes in appearance after 3 d; Daikenchu-tou Kampo extract formulation showed obvious changes in appearance after 7 d; Belsomra Tablets 20 mg and Micardis Tablets 40 mg showed no changes in appearance when not stored in moisture-suppression bags. When these medications were stored in moisture-suppression bags, any changes in the appearance of the medications were not as apparent as when moisture-suppression bags were not used for storage (Fig. 5).

## Discussion

The survey results indicate the status of one-dose package dispensing among community pharmacists in Chiba. When medications are unsuitable for one-dose package dispensing, measures, such as attaching PTP sheets of the medications to the one-dose packages, are undertaken^4^. If these currently adopted measures are effective, few pharmacists would encounter the problem of moisture absorption. However, the results of the survey suggest that many pharmacists have encountered this issue and experienced situations in which they were forced to pack hygroscopic medications in a one-dose package. In fact, many respondents desired one-dose packaging to improve patient compliance and adherence and reduce the burden of dispensing. One-dose packaging of hygroscopic medications has the potential to resolve these issues in clinical practice.

Few studies have evaluated the stability of hygroscopic medications after one-dose packaging, which is an issue in clinical practice. The strength of the current study is that it evaluated the stability of medications for which one-dose packaging is desired in clinical practice. The stability of medications can be maintained by preventing moisture absorption. However, Kampo extract formulations showed marked weight changes even when stored in moisture-suppressing bags. The Kampo extract formulation, Yokukansan, can be stored in a plastic bag with a zipper that contains a desiccant; however, the specific amount of desiccant to be used has not yet been reported^12^. Based on the results of the present study, there is scope for further consideration regarding the type and amount of desiccant required.

For Belsomra Tablets 20 mg, the breaking force was reduced to some extent because of moisture suppression. The tablets behaved differently to other medications, with breaking force increasing with moisture absorption and decreasing with moisture reduction. This may be due to the deliquescence effect of copolyvidone, which is an additive in the Belsomra Tablets 20 mg^13^. The appearance of the Belsomra 20 mg tablets and the Kampo extract formulations, which showed weight changes after moisture-suppression bags were used, was unchanged compared to that when the drugs were stored without moisture-suppression bags. The results of the current survey indicate the difficulty in judging the quality of hygroscopic medications by changes in appearance alone, especially when they are stored in one-dose packaging. In this survey, drugs for which there were high numbers of responses indicating a desire to be packaged in a one-dose package tended to be those that had numerous reports of moisture absorption. Potassium l-aspartate and sodium valproate tablets, which were among the top three products for which there were the highest numbers of reports of moisture absorption in this survey, showed changes in appearance due to moisture absorption after day 7^8^. Additionally, the Bufferin Combination Tablet A81 showed changes in appearance due to moisture absorption after 3 d. In contrast, only one case of moisture absorption was reported in suvorexant tablets, for which the second highest number of responses indicating a desire for one-dose packaging was received. These results suggest that no change in appearance was observed after one-dose packaging for suvorexant tablets and that one-dose packaging may have been continued without moisture absorption being noted. In fact, a survey of hospitals revealed that Belsomra tablets are dispensed in a one-dose package^14^. Thus, for drugs whose quality cannot be judged by their appearance, it is necessary to take measures such as providing guidance on appropriate storage methods.

The results of the survey indicate that community pharmacists mainly referred to medical package inserts and medical interview forms when deciding whether to use a one-dose package. However, medical package inserts and interview forms lack information on the stability of packaged medications under various storage conditions and are inadequate references on which to determine the feasibility of one-dose packaging. For example, the medical package inserts for potassium l-aspartate tablets, for which the greatest number of responses indicating moisture absorption were received in this study, state, “This product is not suitable for one-dose packaging. However, if one-dose packaging is required, the tablets should be stored in a sealed container, and desiccants should be added as needed to prevent moisture absorption.” Yet, the specific type and amount of desiccant to be used is not indicated.

The medical package inserts for Belsomra tablets, for which the second highest number of responses indicating a desire for one-dose packaging were received, states that “tablets should be placed in a PTP sheet, protected from light and moisture, and removed from the PTP sheet immediately before taking.” Under hot and humid conditions, the quality of Belsomra tablets can be maintained for 28 d; however, their weight increased when stored in aluminum bags^15^. The results of the current study were consistent with those of previously reported cases of aluminum bag storage. Despite a slight increase in weight, quality was maintained. However, since aluminum bags cannot maintain a constant humidity, the humidity may increase each time the bag is opened or closed, and may consequently affect the formulation of its contents, depending on the external environment. The humidity control bags used in the current study were designed to maintain a constant level of humidity inside the bags. Thus, the effect of humidity caused by opening and closing the bags was reduced compared to the use of normal aluminum bags. The humidity-controlled bags used in the current study present a clinically appropriate storage method since the actual, single-packaged drug is stored in such a bag for several days, with intermittent opening and closing each time the drug is taken. Therefore, it is necessary to use the most recent information from reliable sources when the information in medical package inserts and interview forms is insufficient. In the current survey, none of the community pharmacists working in insurance pharmacies referred to an article when deciding whether to use one-dose packaging. Therefore, pharmaceutical companies must demonstrate the stability of medications through specific storage methods, and community pharmacists need to improve their information literacy to obtain and use information not only from package inserts and interview forms, but also from the latest research.

Many respondents indicated that improvements should be made to the one-dose packaging material. Therefore, improvements to the storage method and the one-dose packaging paper should be considered. However, a limitation of this study is that, despite the stability observed in the formulation, we did not perform a pharmacokinetic assessment on the tablets, which is necessary for future clinical applications. In the future, we intend to explore better preservation methods by investigating improvements in the one-dose packaging paper. Our findings will aid in addressing issues related to using one-dose packaging in clinical practice.

## Conclusions

The results of the survey indicated the status of one-dose package dispensing among community pharmacists in Chiba. At present, the decision to include hygroscopic pharmaceuticals in a single package based on changes in appearance alone is insufficient. Since one-dose package dispensing may be possible depending on the method of storage, pharmaceutical companies should provide appropriate information, and the information literacy of pharmacists should be improved.

## Data Availability

The data that support the findings of this study are available from the corresponding author upon reasonable request.

## Abbreviations

PTP: Press-through package
RH: Relative humidity
